# An Aesthetic and Economic Approach of Smile Designing for a Patient With Dentinogenesis Imperfecta: A Rare Case Entity

**DOI:** 10.7759/cureus.53978

**Published:** 2024-02-10

**Authors:** Arunoday Kumar, Babina Chirom, Rajesh Nongthombam, Thingujam Debica, Braj Mall

**Affiliations:** 1 Department of Prosthodontics and Crown & Bridge, Dental College, Regional Institute of Medical Sciences, Imphal, IND; 2 Department of Oral and Maxillofacial Surgery, Dental College, Regional Institute of Medical Sciences, Imphal, IND

**Keywords:** smile designing, porcelain fused to metal (pfm) crowns, vertical dimension of occlusion (vdo), pankey mann schuyler (pms), dentinogenesis imperfecta (dgi)

## Abstract

This is a case report presenting a female patient in her twenties suffering from severely stained, unaesthetic, and worn-out teeth since her childhood. It was a major aesthetic and functional concern for her. This clinical presentation describes the prosthetic rehabilitation of a patient with generalized discolored and worn-out teeth to have enhanced aesthetics and masticatory function of the patient. This is a referred case of dentinogenesis imperfecta- II (DGI-II) from the Department of Oral Medicine and Radiology and Oral Pathology, as diagnosed by them after a thorough clinical, radiographical, and histopathological examination.

DGI is a disorder of teeth characterized by discoloration and rapid wear and belongs to a group of disorders of the development of teeth. Due to the rapid wear and generalized intrinsically stained and discolored teeth, there is a loss of vertical dimension of occlusion (VDO) and an unesthetic look of the patient respectively. Therefore, the main objective of the case report is to re-establish the aesthetic and regain the VDO and functionality of the damaged teeth using the Pankey Mann Schuyler philosophy in which the first anterior teeth were rehabilitated with porcelain fused to metal (PFM) crowns based on aesthetics and phonetics of the patient. This was followed by posterior PFM crowns based on Broadrick’s flag analysis for posterior occlusal plane determination and centric occlusion.

## Introduction

Dentinogenesis imperfecta (DGI) is a genetic disease in which teeth development is disrupted. It is characterized mainly by dentine abnormalities resulting in yellowish-brown or blue-gray and translucent teeth [1]. The teeth are weaker than normal thus making them prone to rapid wear, breakage, and loss. Sometimes the teeth look amber or gray because the enamel chips out very soon after their eruption in the oral cavity. DGI is caused by mutations in the dentin sialophosphoprotein (DSPP) gene [2]. DGI affects both deciduous and permanent teeth and shows an autosomal dominant mode of inheritance. DGI can be divided into three types according to the clinical and systemic presentation. Type I DGI is characterized by discolored and brittle teeth along with systemic manifestations of brittle bones (osteogenesis imperfect). At the same time, the sclera of the eyes would be blue. Primary teeth are affected more than permanent teeth. Patients with DGI type II are not affected by osteogenesis imperfecta but exhibit other clinical signs and symptoms [3]. Primary teeth and permanent teeth are equally affected. Type III DGI is characterized by rapid erosion of the primary and permanent teeth. The dental pulp may be exposed. The pulp may be opalescent, smooth, or amber-colored. The survey and review of literature studies on DGI-III conclude that this disorder is limited and restricted to the population of Brandywine in southern Maryland [4]. Prosthetic rehabilitation for all types of DGI aims to restore teeth, aesthetics, and functionality through the steps involved in smile designs and occlusal plane analysis. Smile design aims to provide an aesthetic appearance with natural dentition using several interdepartmental treatment approaches such as orthodontic, periodontal, conservative endodontic, or prosthetic rehabilitation approaches.

In this case report, full mouth rehabilitation of the DGI-II patient with porcelain fused to metal (PFM) crowns and bridges following the Pankey Mann Schuyler (PMS) philosophy is used to restore the teeth to their natural form, function, and aesthetics. This would improve the patient’s oral health, quality of life, and social well-being. By the final result of this treatment, these aims and objectives were fulfilled.

## Case presentation

**Figure 1:**
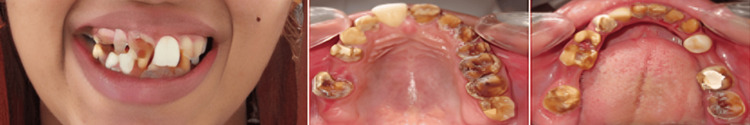
Pre-operative extra-oral and intra-oral view

This is a clinical case of a female patient, in her twenties who reported to the Department of Prosthodontics and Crown & Bridge (referred from the Department of Oral Medicine & Radiology and Oral Pathology). She had complained of severely stained and worn-out teeth, difficulty eating food, and poor aesthetics (Figure 1).

She had not provided any significant medical history of any kind of medicine-specific allergy and her physical growth was normal according to her age. Oral examination confirmed that she did not have any kind of deformity, pain, or crepitus in the temporomandibular (TMJ) joints or any other bones of her face. The TMJ was well coordinated and had bilateral symmetrical movements [5]. The intraoral examination showed that she had yellowish-brown stained-looking teeth with a reduction in the vertical dimension of occlusion (VDO) due to wearing out of the occlusal surface which is a typical feature of DGI. The patient was diagnosed with DGI-II after clinical, radiographical, and histopathological examination by the Department of Oral Medicine & Radiology and Oral Pathology. The patient had dental caries concerning 11, 12, 16, 24, 44, and 46 teeth and a partial edentulous arch concerning 15, 17, 37, and 45 teeth.

After all the dental conditions of the patient were examined, an interdepartmental approach was used to perform full-mouth rehabilitation according to the PMS philosophy. The whole treatment process can be viewed in four steps. The first step includes proper examination, diagnosis, and treatment planning. The second step is the determination of anterior incisal guidance to provide the best smile to the patient. The third step included planning and selecting the best and most comfortable occlusal plane. The final step is the restoration process, which is required to ensure good intercuspation between the upper and lower posterior teeth [6].

The clinical condition of the available crown and its periodontium was evaluated for its optimum health to support the prosthesis. Radiographical examination of individual teeth and evaluation of available inter-arch space in jaw relation were done to do treatment planning for smile designing following PMS philosophy. In PMS philosophy, first prosthetic rehabilitation for mandibular anterior teeth was done followed by rehabilitating the maxillary anterior teeth based on the best aesthetics and phonetics which would be in harmony with the incisal guidance and condylar guidance of the patient. This would be followed by prosthetic rehabilitation of mandibular posterior teeth as per the best acceptable occlusal plane verified by Broadrick’s occlusal flag analysis, which would be in harmony with the incisal and condylar guidance of the patient. Finally, prosthetically rehabilitating the maxillary posterior teeth so as to have maximum intercuspation in centric and canine-guided occlusion in eccentric jaw movements.

**Figure 2:**
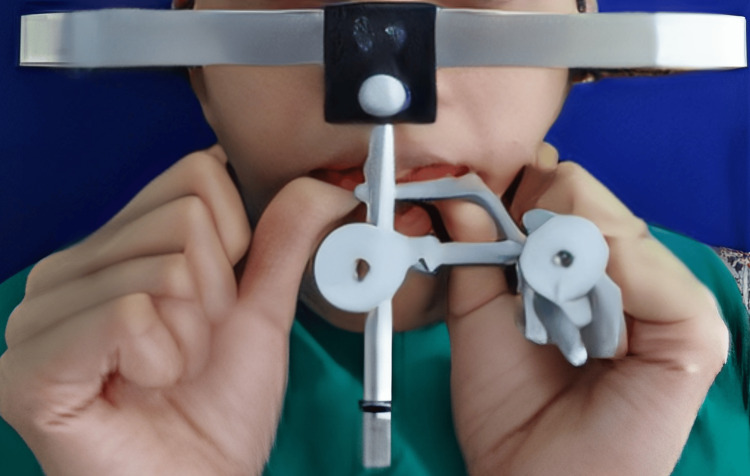
Diagnostic mounting using face bow transfer

**Figure 3:**
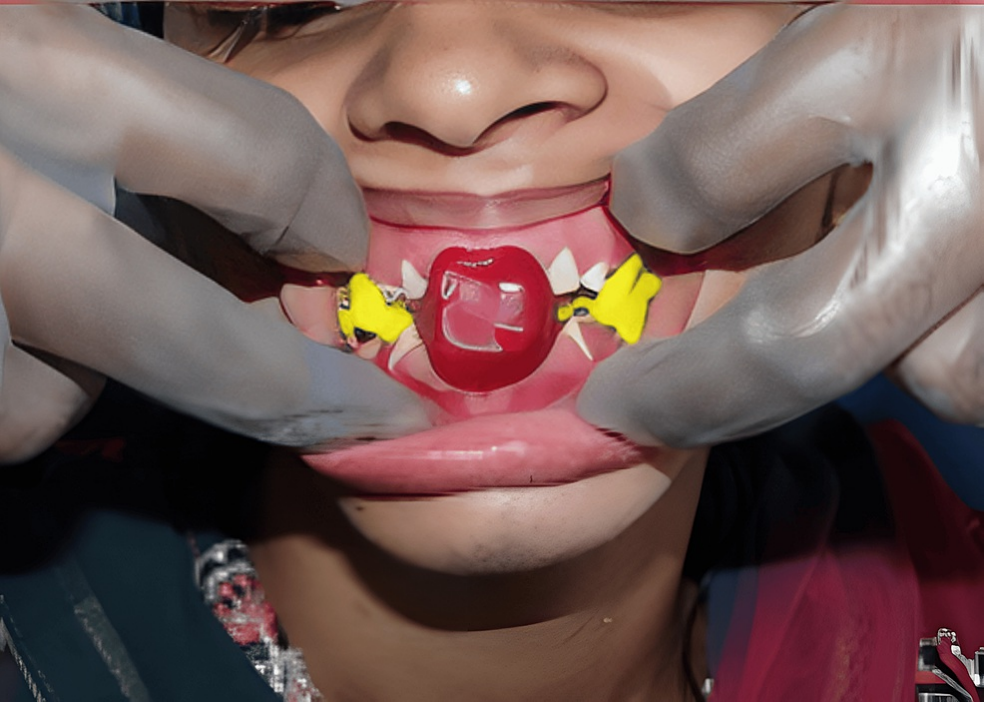
Anterior jig and posterior interocclusal check record of 4mm thickness in centric relation

Treatment was planned in a way so that oral prophylaxis, extraction of 48, endodontic therapy of 11, 12, 16, 24, 44, and 46 teeth, and crown lengthening of the maxillary anterior teeth preceded the prosthetic rehabilitation by increasing the VDO by 4 mm from the existing collapsed bite [7]. Raising the VDO by 4 mm has to be done to regain natural freeway space of 4 mm, as the patient had 8 mm of existing freeway space. The Hanau Wide Vue articulator and the face bow transfer (Figure 2) were used for diagnostic mounting at a raised VDO of 4 mm from the existing collapsed bite, with the help of a 4 mm thick posterior interocclusal check record and anterior jig (Figure 3).

**Figure 4:**
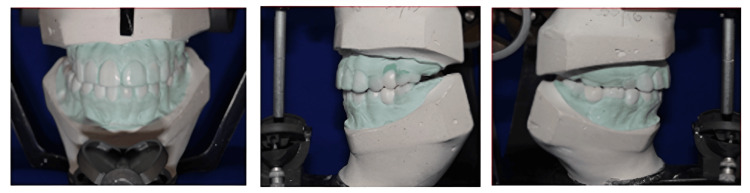
Frontal view, left lateral view, and right lateral view of wax mock-up after diagnostic mounting at raised VDO of 4mm VDO: Vertical dimension of occlusion

To restore the anterior aesthetics, phonetics, anterior incisal plane, posterior occlusal plane, and centric occlusion between the upper and lower posterior teeth, the diagnostic wax mockup was done as per the harmonious relationship between the anterior and condylar guidance of the patient. Broadrick’s occlusal flag analysis is used for wax mock-up for the mandibular posterior teeth (Figure 4).

Broadrick’s flag analysis for the determination of the mandibular occlusal plane was performed with the help of a caliper which was set at a radius of 4 inches from the needlepoint to the pencil point. The needle point of the caliper was set against the canine tip and was scribbled on the flag (anterior survey line). The caliper point was held against the condylar ball of the articulator and another arc was in the fag (condylar survey line). From the intersection point, a line is drawn from the molar to the canine. Therefore, an acceptable occlusal plane for the mandibular posterior teeth was obtained. 

**Figure 5:**
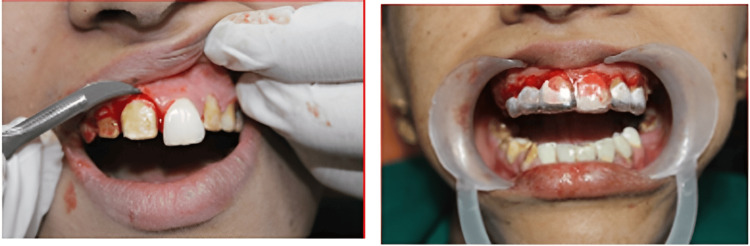
Gingivectomy and surgical stent for crown lengthening to convert gummy smile to papillary smile

Gingivectomy with a surgical stent was performed for crown lengthening and periodontal dressing with Coe-Pak was given and allowed to heal for two weeks (Figure 5).

**Figure 6:**
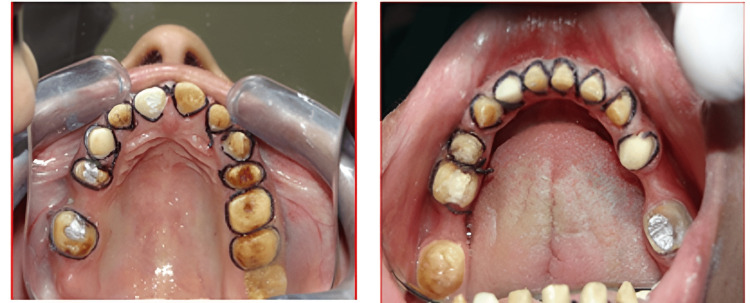
Maxillary and mandibular tooth preparation and gingival retraction cord placement

A healthy contour of the gingiva is seen after two weeks of post-gingivectomy (Figure 6). Tooth preparation was done in the same appointment, as recommended for PFM crowns. Minimal occlusal tooth preparation was done as the teeth were already worn out due to the existing condition of DG-II. The proper gingival retraction was done with the gingival retraction cord and gingival packer (Figure 6).

**Figure 7:**
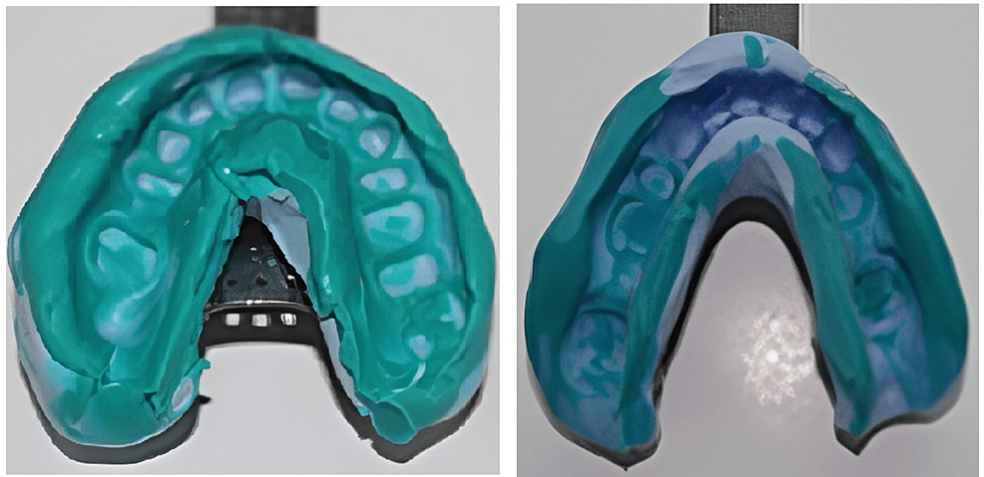
Maxillary and mandibular final impression with the double mix and double impression technique

The final impression of the maxillary and mandibular prepared tooth was made with putty and light body consistency addition of silicone elastomeric impression material, following the double mix double impression technique (Figure 7). The final impression was poured with die stone to get the master cast.

**Figure 8:**
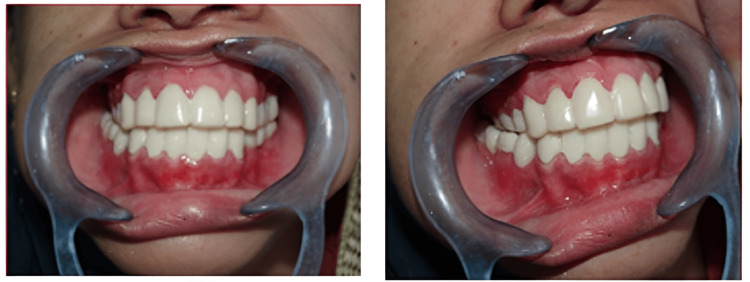
Frontal view and lateral view for temporization of the prepared tooth

The final impression was poured with die stone to get the master cast. Provisional crowns using Protem^TM^ Plus as a temporary restorative crown material were fabricated with the help of putty index (made from the diagnostic Wax mock-up cast) following the direct technique of its fabrication (Figure 8).

**Figure 9:**
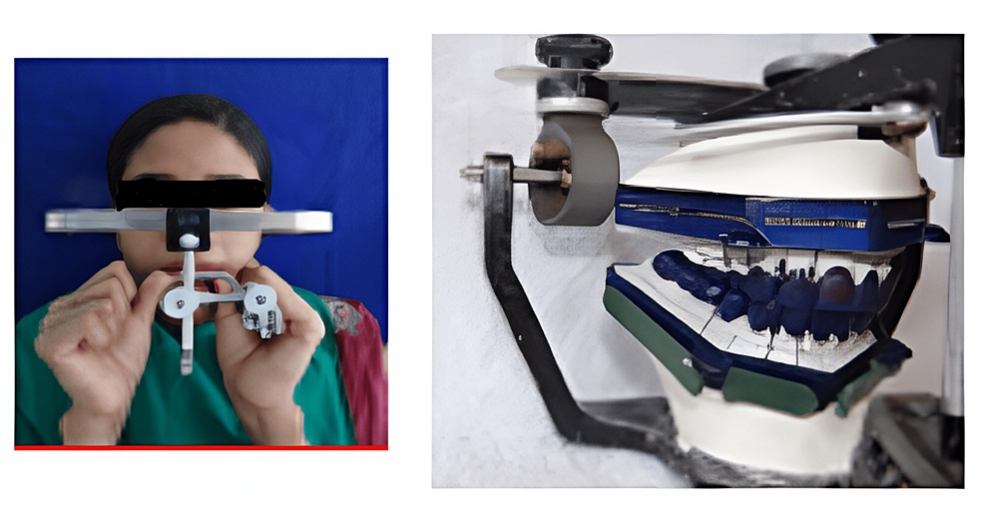
Mounted master cast onto Hanau Wide Vue articulator with inlay pattern wax preparation with the help of face bow transfer

Provisional crowns were thus temporarily cemented with zinc oxide eugenol (ZOE) temporary cement, onto the prepared teeth and kept for four weeks to look for any TMJ discomfort or masticatory muscle spasm as it was made at 4 mm of raised VDO. The patient was also assessed for esthetics, phonetics, and functional harmony. As the patient did not report any TMJ or muscular spasm even after four weeks of temporization or provisional restorations, the final prosthesis was planned with full mouth prosthetic rehabilitation, having canine-protected occlusion using PMS philosophy. This philosophy was used to obtain anterior guidance based on aesthetics and phonetics. The posterior occlusion should be in harmony with the incisal guidance and condylar guidance of the patient in centric with maximum intercuspation and disocclusion on protrusion and lateral excursive movements. The maxillary master cast was mounted to the upper member of the articulator with the help of face bow transfer (Figure 9). The mandibular cast was mounted to the lower member of the articulator with the help of provisional crowns and an interocclusal check record. 

After mounting the master cast, the temporary crowns were re-cemented with ZOE temporary cements onto the prepared teeth till the permanent PFM crowns were fabricated in the laboratory.

Inlay pattern wax is used to fabricate the individual wax pattern copings following the cut-back technique (facially and buccally). The cut back technique for wax copings was used to have the ceramic facing in the facial and buccal aspects (Figure 9).

**Figure 10:**
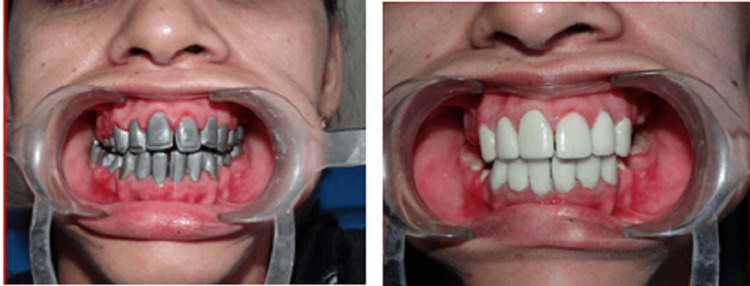
Metal copings try in and cementation of the anterior prosthesis after ceramic build-up

The wax pattern was metal cast, finished polished, and finally tried in the patient’s mouth to ensure precise fit onto the margins of prepared teeth (Figure 10).

The metal copings were re-fitted onto the master cast which was mounted onto the semi-adjustable articulator and ceramic facing was done in two phases. In the first phase, the porcelain build-up was done with facing in the maxillary and mandibular anterior teeth making sure that maxillary anterior teeth made contact on protrusion and canine making contact on lateral excursive movement in the working side (canine guided occlusion). This made the anterior guidance to be established for the patient depending on the patient’s aesthetics and phonetics.

The maxillary and mandibular anterior prosthesis was permanently cemented with Fuji 1 GIC (Glass Ionomer cement) Type I, onto the prepared tooth. As the posterior teeth are already prepared, the final impression is once again made with elastomeric impression material and subsequently poured to get a master cast.

**Figure 11:**
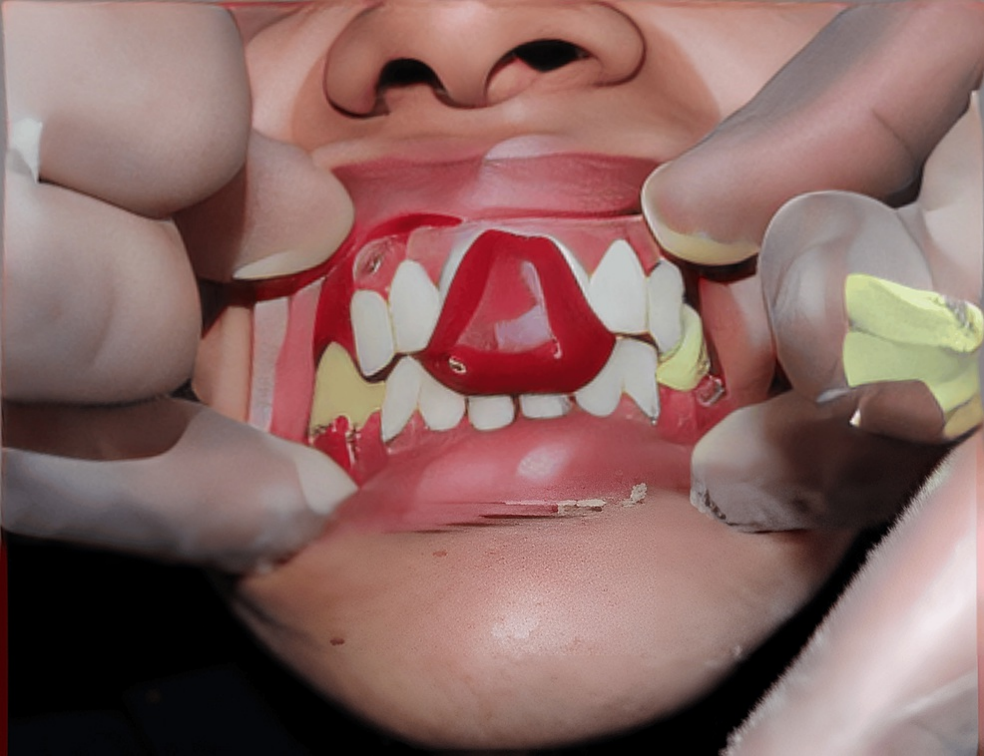
Anterior jig and posterior interocclusal check record in centric, at raised VDO of 4 mm after permanent cementation of anterior prosthesis VDO: Vertical dimension of occlusion

It was followed by fabricating an anterior jig and posterior interocclusal check record for mounting the newly fabricated master cast (positive replica of the anterior permanent prosthesis with posterior prepared teeth) to the semi-adjustable articulator with the face bow transfer (Figure 11).

**Figure 12:**
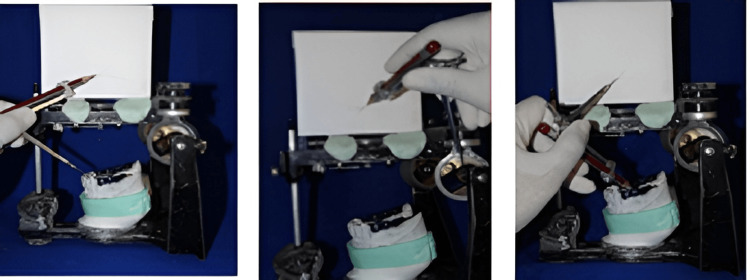
Broadrick occlusal plane analyzer for the posterior occlusal plane determination

After mounting, the posterior occlusal morphological structure was built up with the inlay pattern wax and reverified with Broadrick’s occlusal flag analysis, which was done at the diagnostic wax mock-up (Figure 12). This was followed by a cut-back of inlay pattern wax in the buccal aspect, which provided the provision for porcelain build-up in the buccal aspect of the crown.

**Figure 13:**
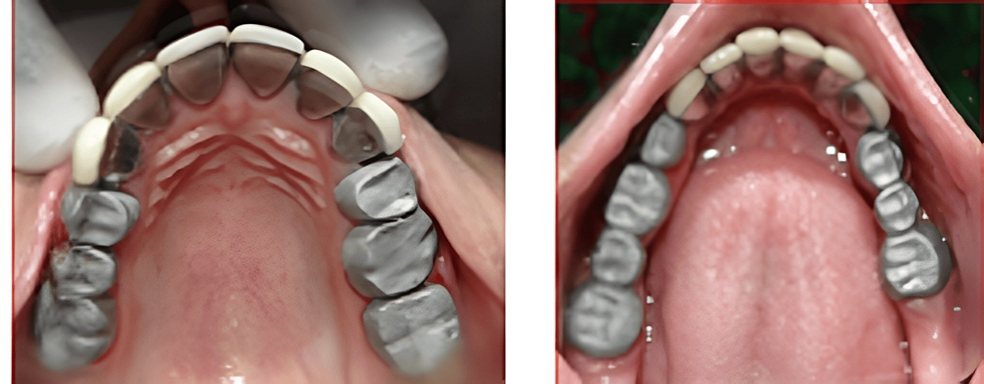
Posterior metal coping try-in

**Figure 14:**
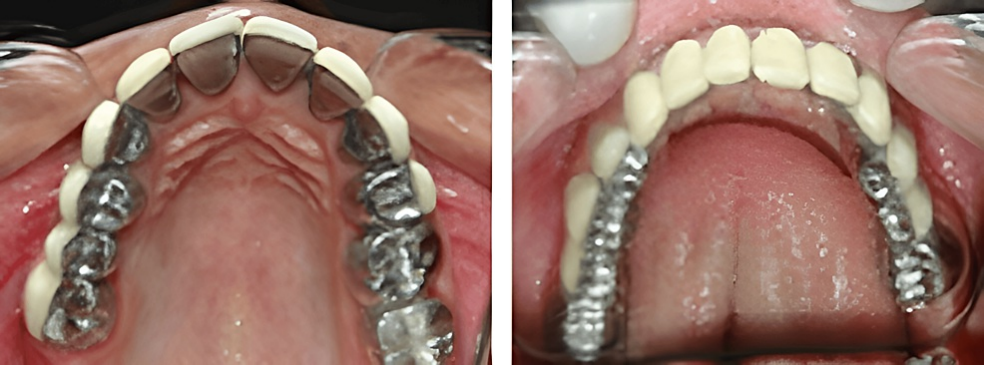
Cementation after porcelain facing build-up

After casting of the inlay pattern, metal try-in of the posterior teeth was done and the buccal facing of the metal cast was built up with ceramic (Figure 13 and Figure 14) to re-contour the morphology of the posterior crowns and was re-verified with the Broadrick’s occlusal flag analysis. 

Porcelain facing is used to prevent the clicking sound that is heard when one ceramic crown comes in contact with an opposing ceramic crown and is very annoying for the patient. Also, the metal crowns are more durable as the ceramic or porcelain crowns tend to wear or chip out in the long run.

**Figure 15:**
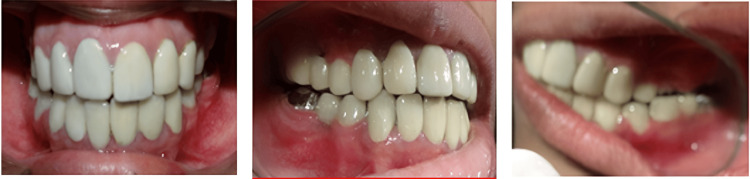
Frontal, right lateral, and left lateral view of intra-oral post-cementation of the prosthesis in centric relation

**Figure 16:**
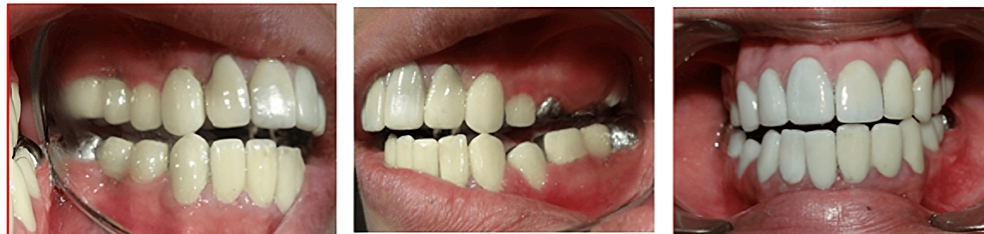
Lateral and frontal views of posterior disocclusion and canine-guided occlusion in eccentric movements (lateral and protrusive)

Before cementation of the permanent prosthesis, the centric occlusion was verified and the posterior disocclusion of left and right posterior teeth was achieved on lateral excursive movement and only canine coming in contact which is canine-guided occlusion (Figure 15 and Figure 16).

**Figure 17:**
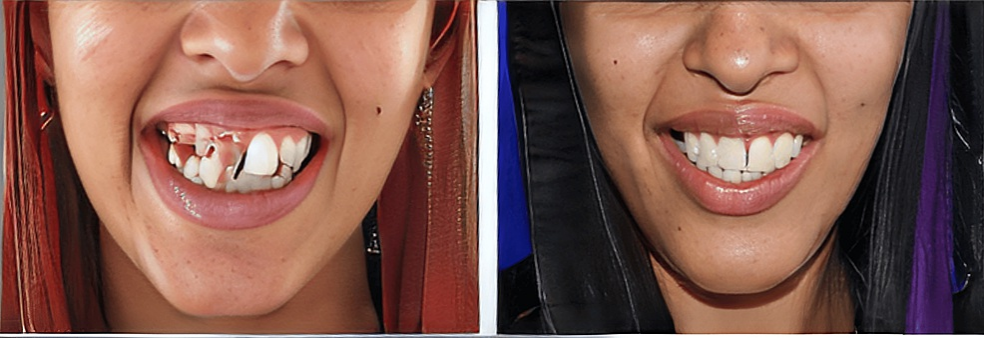
Pre-operative and post-operative comparative evaluation aesthetically (post-cementation)

The occlusion was adjusted to have any interferences removed by selective grinding and finally, the PFM crown was glazed and permanently cemented using Fuji 1 GIC (Glass Ionomer Cement) Type I cement (Figure 17) onto the prepared teeth, to have proper function, aesthetics, and phonetics.

The patient was given oral hygiene instructions to maintain good oral hygiene and regular follow-up was scheduled for a good prognosis of the prosthesis.

## Discussion

DGI is a rare disorder characterized by dental manifestations in the form of discolored and worn-out teeth which leads to a reduction in VDO thereby leading to an unaesthetic appearance with reduced chewing efficiency. For full-mouth rehabilitation, various occlusal schemes can be found in the literature including the PMS technique, Hobo’s twin table, Hobo’s twin stage, etc.

In Hobo’s twin table technique, occlusal anatomy of the posterior teeth is replicated based on the condylar guidance record of the patient, subsequently, replication of anterior anatomy of teeth based on anterior guidance. The disadvantage of this technique is that the patient is not comfortable as the steep posterior cuspal angle makes the incisal guide table be set at a steep angle too, which can affect the morphology of anterior teeth. However, in Hobo’s twin-stage technique, the occlusal anatomy is replicated based on the standard cusp angle and therefore the record of condylar guidance from the patient is not required. Anterior anatomy of the crown is made depending on acceptable incisal guidance, which would fit best as per esthetics phonetics, function, and comfort of the patient. The limitation of Hobo’s twin-stage technique is that it cannot be used for maloccluded teeth.

No philosophy can be said to be universal, however, among these procedures, PMS and Hobo’s twin stage are widely used techniques [8]. The PMS philosophy is the most used as stated by Thimmappa et al. [9]. In this work, the full mouth rehabilitation of patients with worn-our teeth was done with both the techniques by grouping the patients, and its impact on the oral health of the patients was studied.

Also, a research article by Prakash et al. which was an in vivo study, assessed the impact of these two rehabilitation philosophies (PMS and Hobo-twin stage) in 40 patients’ oral health [10]. This study showed that the PMS philosophy resulted in optimum occlusion in terms of maintaining the patient’s VDO and establishing the perfect occlusal plane.

Also, a case report by Kumar et al. in 2023 November, elaborated on a similar technique used for full mouth rehabilitation of amelogenesis imperfecta patients [11]. This article explains the prosthodontic rehabilitation of all the affected teeth, for Amelogenesis Imperfecta patients using PMS Philosophy, in which mandibular and maxillary anterior teeth were rehabilitated with all ceramic crowns followed by rehabilitation of mandibular and maxillary posterior teeth with PFM crowns with buccal facing.

In this case report, we used the PMS philosophy to do the needful treatment for full mouth rehabilitation. The PMS technique is preferred over Hobo’s because it is a simple, well-organized acceptable technique that progresses smoothly with less strain on the operator, patient, and technician [12]. 

Regaining back the aesthetics, phonetics, and masticatory function of the patient by rehabilitating all the affected teeth with PFM crowns having only ceramic facing is an economical and aesthetic approach. PFM crowns with only ceramic facing are of less cost with optimum aesthetic outcome compared to that of the porcelain jacket crowns and all ceramic crowns which are costly and cannot be afforded by the economically weaker population of the society [12].

This case of DGI-II was prosthetically rehabilitated with PFM crowns with ceramic facing in all the affected teeth for smile designing [13]. This treatment of smile designing with full mouth rehabilitation using PMS philosophy had a good prognosis as the patient reported with the healthy condition of periodontium even after six months of follow-up. The only limitation of this technique was that the procedure was time-consuming as the procedure was completed in several short appointments [14].

## Conclusions

This case reports a DGI-II patient with severely stained, worn-out anterior and posterior teeth and a reduced VDO by 4 mm (collapse bite) which hampered the patient’s chewing capacity and adversely affected the functionality of the teeth and the aesthetics. To treat, an interdepartmental approach was planned which included endodontic therapy, gingivectomy for crown lengthening, and prosthetic rehabilitation for smile designing with PFM crowns and bridges following the PMS philosophy. The treatment procedure was easy and comfortable for the patient as it was completed in several short appointments. In addition, the PFM crowns with facing are an economical approach to smile design. This treatment outcome had a beautiful smile on the patient thereby enhancing aesthetics, phonetics, and masticatory function.
